# Application of career related research in Pakistan: The case of apples vs mangoes

**DOI:** 10.12669/pjms.323.9864

**Published:** 2016

**Authors:** Zarrin Seema Siddiqui

**Affiliations:** Dr. Zarrin S. Siddiqui, PhD. Education Centre, The University of Western Australia, Perth, Australia

**Keywords:** Career choice, Medical students, Specialty preference

## Abstract

Career choice of medical graduates is dependent on a number of factors as identified in literature across the globe. This article presents an overview of contemporary research on career choices of medical graduates and the generalisation of findings in Pakistan. A number of issues are highlighted which include lack of standardised instruments, classification of specialties and mixed evidence regarding variables and socio cultural differences..

## INTRODUCTION

Pakistan is facing a number of issues concerning the medical workforce which include lack of specialists in various specialties, migration and leaving the profession altogether hence there is a need to determine the career choices of medical graduates to provide evidence based recommendations. While literature identifies a range of influencing factors across the globe, these results have certain implications for application in Pakistan. In this article, an overview of contemporary research in the area is presented with implications for Pakistan.

### Classification of Specialty Groups

There does not appear to be one classification of specialty groups among researchers and it varies from one study to another. The various ways in which specialties have been classified to research career choices are:

- Technique oriented vs. person – oriented specialties:
  Technique oriented specialties are the ones that deal with techniques and instruments such as Surgery compared to person–oriented which are more oriented towards people.^1^,^2^
- Primary care vs. non primary care specialties:
  Primary care specialties include General Internal Medicine, Paediatrics and Family Medicine, while the rest specialties are grouped in the non-primary care group.^3^-^6^
- iii. Clinical vs. basic medical specialties:
  Basic medical specialties are Anatomy, Physiology, Biochemistry and Pathology which do not require direct patient contact.^7^
- iv. Hospital based vs. Primary care and Technical specialties:
  One study grouped specialties into three groups. The first group comprised primary care specialties as described earlier. The second group included hospital-based specialties (Anaesthesiology, Emergency Medicine, Pathology, Radiology), while the third group included technical and surgical specialties (Neurology, Obstetrics and Gynaecology, Ophthalmology and all surgical fields) and found significant differences on various factors.^8^
- v. Congruent vs. non congruent specialties:
  This classification is based on an inventory “Medical Specialty Preference Inventory” (MSPI) that assists students to determine the right specialty. Congruence describes the relationship between one’s actual specialty choice versus the one that was suggested based on the MSPI. It is noted that physicians with congruent specialty choice had higher job satisfaction than those who were not working in the predicted specialty.^9^
- vi. Controllable vs. Non-controllable lifestyle specialties:
  Controllable lifestyle specialties are the specialties where a specialist can control the number of hours devoted to the specialty.^10^ A later study however, cautioned against using the term as the authors suggested that this dichotomy may mask important complexities and a new term “lifestyle friendly” was introduced defining lifestyle friendly career as the one that allows leisure time, opportunities to enjoy life outside of work, predictable work hours, time to pursue activities outside of work hours, and family time.^11^
- vii. Frontline care vs. non frontline care specialties:
  In this classification cited in a Brazilian study, frontline care (FC) specialties included Internal Medicine, Paediatrics, Obstetrics and Gynaecology and General/Family Medicine while all other specialties were included in the non-frontline care specialties^12^

The above mentioned classifications are difficult to generalise because of the different nature of practice in Pakistan. For example, the definition for primary care and non-primary care cannot be applied to Pakistan. The concept of lifestyle is very different. Family Medicine as a specialty is still evolving. It is a common practice for medical graduates to assume the role of general practitioner soon after graduation and for lay public the difference between two is not clear.

### Influence of variables

Studies provide mixed evidence about the strength of variables. For example, educational debt is a weak variable compared to other influencing factors.^13^,^14^ There is some evidence that in the United States of America (USA) the level of debt affects practice locations,^15^ but the other studies did not support this hypothesis.

Spousal influence was identified as a factor in a study exploring residents’ choice of Emergency Medicine for both genders but of a lesser influence.^16^ On the other hand parental influence was the third important factor identified a Pakistani study involving medical students.^17^

Stress is another influencing variable,^18^,^19^ but was not perceived to be an important influencing factor for those who selected surgical specialties in Pakistan,^20^ On the other hand general perception of the public about a specialty was the most important predictor in a study of medical students in Pakistan.^17^ which is not found in studies elsewhere.

### Personality types

There are numerous studies which have tried to link the personality type with medical careers. Holland’s theory of career choice relates to personality fit model.^21^ Holland proposed that most people are one of six personality types and among physicians, the most common personality types are the investigative, social and artistic types which was further validated in another study.^22^ The Myers-Briggs Type Indicator (MBTI) is another instrument that has been used to determine the profile of personality types.^23^ This instrument has four dimensions on which the personality profiles are measured. Of those selecting non-primary care are mostly male, extraverted, and thinking types with a preference towards surgical specialties while women who selected non-primary care were in the domain of introverted, and feeling types.^23^

The Sixteen Personality Factor Questionnaire(16PF) was used by Hartung in an attempt to link people in occupations based on identical inventory profiles and in medical specialty preferences the choice was correctly predicted 43-60% of the time.^24^ Attachment theory has also been used to suggest that medical students with secure style were more likely to choose primary care (61%) over non-primary care when compared to students whose styles were characterised by self-reliance, support-seeking or caution who chose primary care (41%).^5^

The German Extended Personal Attributes Questionnaire (GE-PAQ) and Career motivation questionnaire were also used in a Swiss study to correlate the career choices of medical students.^25^ Female students scored higher on traits such as helpfulness, relationship consciousness, and empathy as well as on extra professional concerns such as family responsibilities, convenient working hours, and job security. On the other hand male students scored higher on independency, decisiveness, self-confidence and activity. When compared to females, they also scored higher on the scale which include striving for promotion, income, and prestige and these results were also reflected their specialty choices.

In a study in 2012, the revised version of NEO Five-Factor Inventory called NEO Personality Inventory (NEO-PI-R) was used to create a profile of women who are interested in Surgery and compare it with their male counterparts and with those women who did not show an interest in Surgery.^26^ It was observed that although less women than men wanted to specialise in Surgery, the ones who were inclined were different from their male counterparts placing greater emphasis on helping people than prestige and lifestyle. This group of women was also different from the women who were not interested in Surgery, and had higher Agreeableness scores placing more importance on prestige than lifestyle.

Another study in the USA investigated combined influence of interests, personality and values in a sample of second year medical students from one medical school using three instruments. These instruments were Strong Interest Inventory (SSI) based on Holland’s personality types, NEO Personality Inventory-Revised (NEO-PI-R), and the Physician Values in Practice Scale (PVIPS).^27^ Interesting observations were made by the researchers in this study suggesting that intellectually curious students with vivid imagination are more likely to pursue careers in medical specialties, such as Psychiatry or Plastic Surgery. Another study of medical graduates used five different inventories to assess personality attributes and if there are any differences between graduates who select General Medicine versus medical subspecialties.^28^ Trends were found towards authoritarians and negative orientation towards patients with psychological problems and Machiavellianism in both groups.

If we look at the limitations of these studies, there are inconsistencies of results as described in the review undertaken by Borges and Savickas in 2002.^29^ The bias resulted from the convenience sampling in these studies may also be one limitation in interpretation of the results while none of the personality inventories used in these studies are validated in Pakistan.

### Standardised instruments

The instruments that are used in the studies are varied except in USA and United Kingdom where standardised instruments have been used over a period of years to asses or observe any shifts and trends in the career decisions of students.

### Respondents

Literature supports that students keep on changing their choices, so the validity of research conducted immediately after a clinical rotation, or when students have not been exposed to all rotations is doubtful. One interesting observation was reported in a study of Irish students where the author reported that students’ clerkship experiences affected their choice of Psychiatry as a career. The same phenomenon was observed in the preceding Obstetrics and Gynaecology rotation.^30^

There are other preoccupations that may not give students enough time to think about their future careers during studies such as assessments. Similarly no differences in the first choice of specialty was found for first year students across five medical schools in Scotland when asked about their career intentions,^31^ or for third year students across four medical schools in Pakistan.^32^

### Influence of motives to enter the medical school

Although a number of studies have investigated the motivation to enter medical course, there is only one Norwegian study that has tried to establish relationship of motivation to become a doctor with gender and specialty preferences.^33^

### Theoretical frameworks

There is lack of sound theoretical frameworks that can be used to research career intentions. A number of decision making theories are reported in the literature but somehow these are not applied in career choices for medical graduates. One possible reason may be that most of these theories explore decision making related to a particular career while the specialty preference of doctors is a different phenomenon as it assesses the second stage of career decision where a cohort has already decided to become doctor and is now investigated to see which pathway is subsequently taken within the chosen career.

## DISCUSSION

If we examine the career related research from the perspective of Pakistan there are several implications. The socio-cultural scenario in Pakistan is different from the reported studies and a number of factors are irrelevant. For example, debt is not an influencing factor as there is no such concept in the Pakistani education system although it is quite expensive to study medicine. Similary malpractice liability is again not a burning issue for practising doctors in Pakistan.

There are few studies from South East Asian countries and this needs to be further investigated within the framework of the cultural and political scenarios in these countries. Similarly, within the reported literature there are inconsistencies between findings and it is difficult to generalise the results to other countries. Another limitation is that none of these studies investigated medical graduates who did not intend to pursue a career in medicine after graduation which is a critical issue in developing countries like Pakistan.

In addition, the entrants to the medical course in Pakistan are high school leavers when compared to graduate students in many of the studies from developed countries who may be more mature and independent in their decision making. Even, when we look at the respondents’ characteristics, most of the studies have used students compared to medical graduates and there is evidence that there are different stresses and priorities for students which may prevent them from making any career decisions during studies.

There are also no studies reported from Pakistan, which have investigated contributing factors to choose a particular medical career until 2006.^34^ Another dimension that has been added to the career choice in recent years is the ethnicity and cultural factors in decision-making. Anecdotally, the ethnicity of the patient population and the area of practice in Pakistan also play a role in determining the career choice because some ethnic groups may not like their women to be seen by a male doctor and vice versa. Similarly Pakistan being a predominant Muslim country the preference of female patients is mainly to be cared for by a female physician.^35^

## CONCLUSION

This overview supports the need for investigation of career intentions among Pakistani medical graduates using a locally developed instrument in order to identify the factors of importance within this region. This information will enable stakeholders to understand the dynamics of current workforce and make relevant policy decisions.
